# Supplementary material for: Harmful R-loops are prevented via different cell cycle-specific mechanisms
Source: Nat Commun. 2021 Jul 22;12:4451. doi: 10.1038/s41467-021-24737-x (PMC8298424; doi:10.1038/s41467-021-24737-x)
Supplement: Supplementary file 1 — Supplementary Information [file 41467_2021_24737_MOESM1_ESM.pdf]

Harmful R-loops are prevented via different cell cycle-specific mechanisms

Marta San Martin-Alonso, María E. Soler-Oliva, María García-Rubio, Tatiana García-Muse * and Andrés Aguilera *

Centro Andaluz de Biología Molecular y Medicina Regenerativa CABIMER, Universidad de Sevilla-CSIC-UPO, 41092 Seville, Spain

Corresponding authors: E-mails: A A: aguilo@us.es
T G-M: tatiana.muse@cabimer.es

SUPPLEMENTARY DATA

Supplementary figures 1-8

Supplementary Tables 1-5

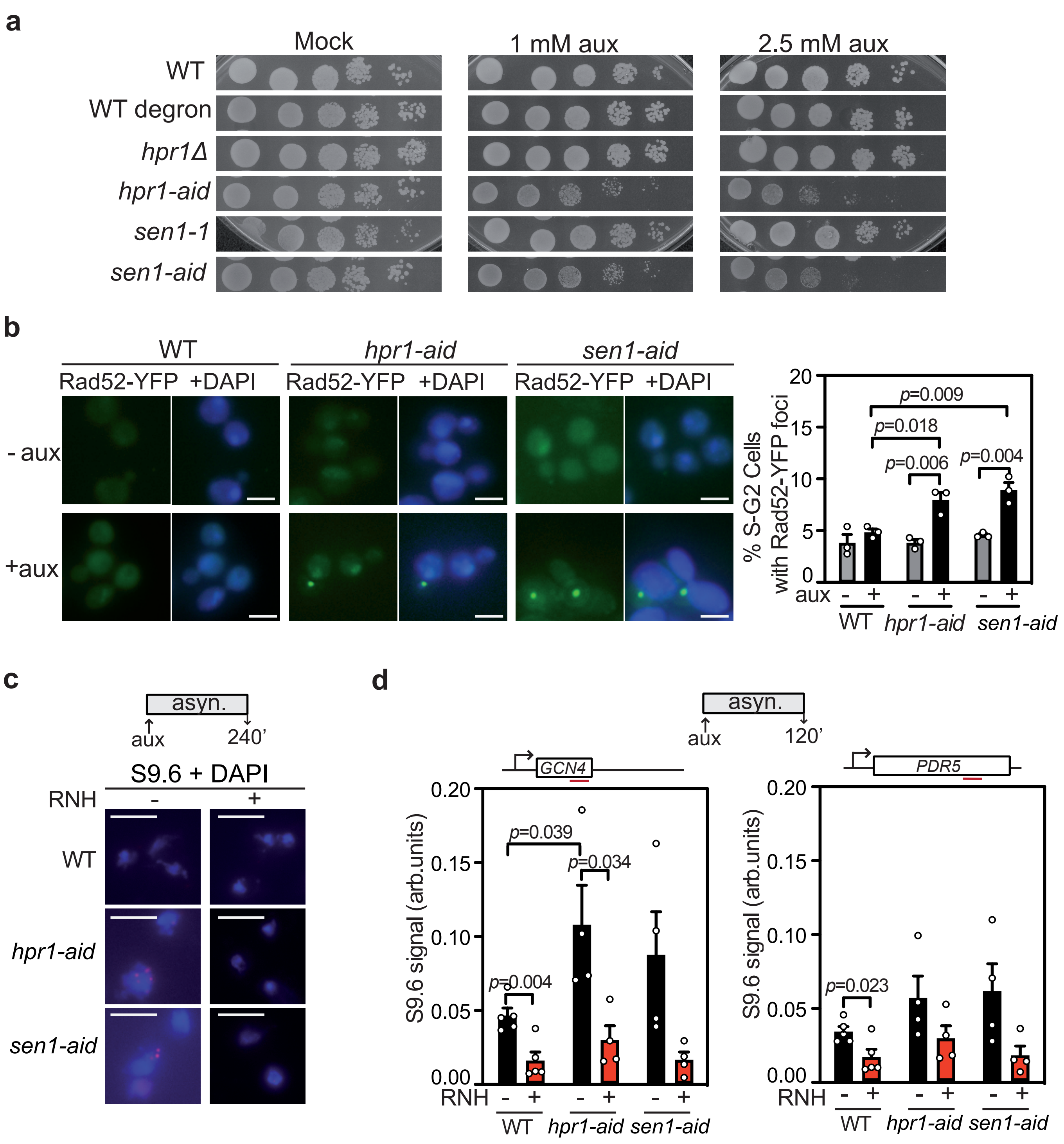

Supplementary Fig. 1. **Fast controlled depletion of Hpr1 and Sen1 using an inducible auxin degron system. a**. Serial dilutions of WT, WT-degron, *hpr1Δ*, *sen1-1* and *hpr1-aid* and *sen1-aid* degron strains in the absence or presence of auxin (IAA). Plates were incubated at 26ºC for 2 days. **b**. Representative images of Rad52-YFP foci formation in *hpr1-aid* and *sen1-aid* strains with or without auxin (n=100 cells examined over 3 independent experiments). Data are presented as mean values +/- SEM. **c**. Representative images of S9.6 foci detected by immunofluorescence on yeast chromosome spreads in WT, *hpr1-aid* and *sen1-aid* asynchronous cells with or without RNH1. **d.** DRIP with S9.6 antibody at *GCN4* and *PDR5* genes in asynchronous cells after 2 hours of auxin addition. WT, *hpr1-aid* and *sen1-aid* were treated (+) or not (-) in vitro with RNH1. Data are presented as mean values +/- SEM (n=4 biologically independent experiments).
A diagram of the experiment is shown at the top of each panel. The P values calculated by the two-tailed unpaired Student t test. Scale bar 5μm. Data underlying this figure are provided as Source data file.

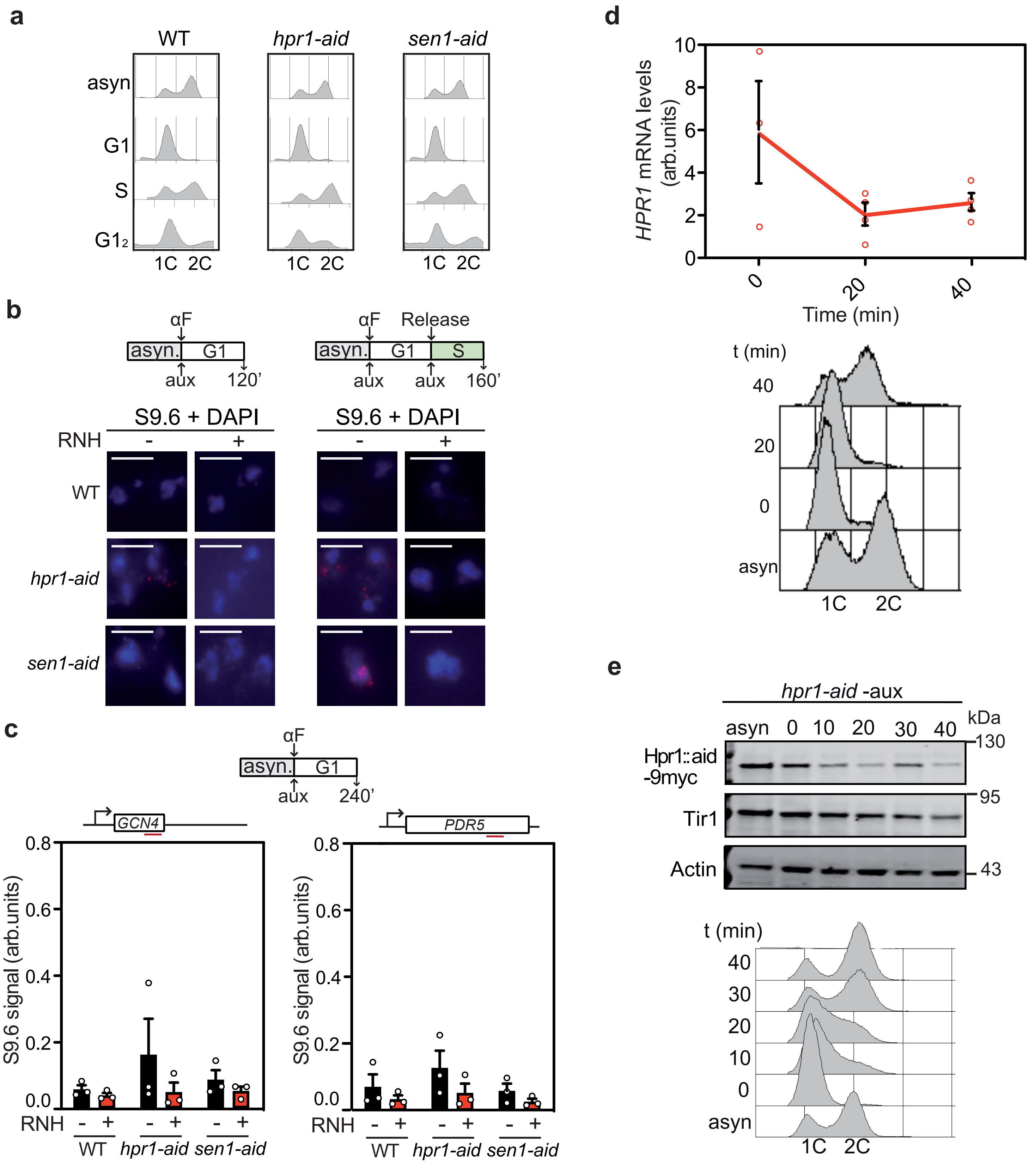

Supplementary Fig. 2. R-loop accumulation after depletion of Hpr1 and Sen1 using an inducible auxin degron system. a. DNA content of experiments shown in b, c and Fig. 3 measured by flow cytometry. A diagram of the experiment is shown at the top of each panel. **b.** Representative images of S9.6 foci detected by immunofluorescence on yeast chromosome spreads in WT, *hpr1-aid* and *sen1-aid* arrested in G1 or in S-phase cells with or without RNH1 of the graphs shown in Fig. 3. **c.** DRIP with S9.6 antibody at *GCN4* and *PDR5* genes in G1-synchonized cells after 4 hours of auxin addition. WT, *hpr1-aid* and *sen1-aid* were treated (+) or not (-) in vitro with RNH1. Data are presented as mean values +/- SEM (*n*=3 biologically independent experiments). **d.** RT-PCR analysis of the *HPR1* gene normalized respect to *SCR1* gene in *hpr1-aid* cells released from α-factor. Data are presented as mean values +/- SEM (*n*=3 biologically independent experiments). Analysis of cell cycle progression after α-factor release of cells is shown below the panel. **e.** Immunoblotting with αHpr1 of protein extracts of *hpr1-aid* cells without auxin in asynchronous cultures and after release from α-factor arrest. AtTIR1-9Myc was used as loading control. Analysis of cell cycle progression after α-factor release of cells is shown below the panel (*n*=2 biologically independent experiments). Scale bar 5μm. Data underlying this figure are provided as Source data file.

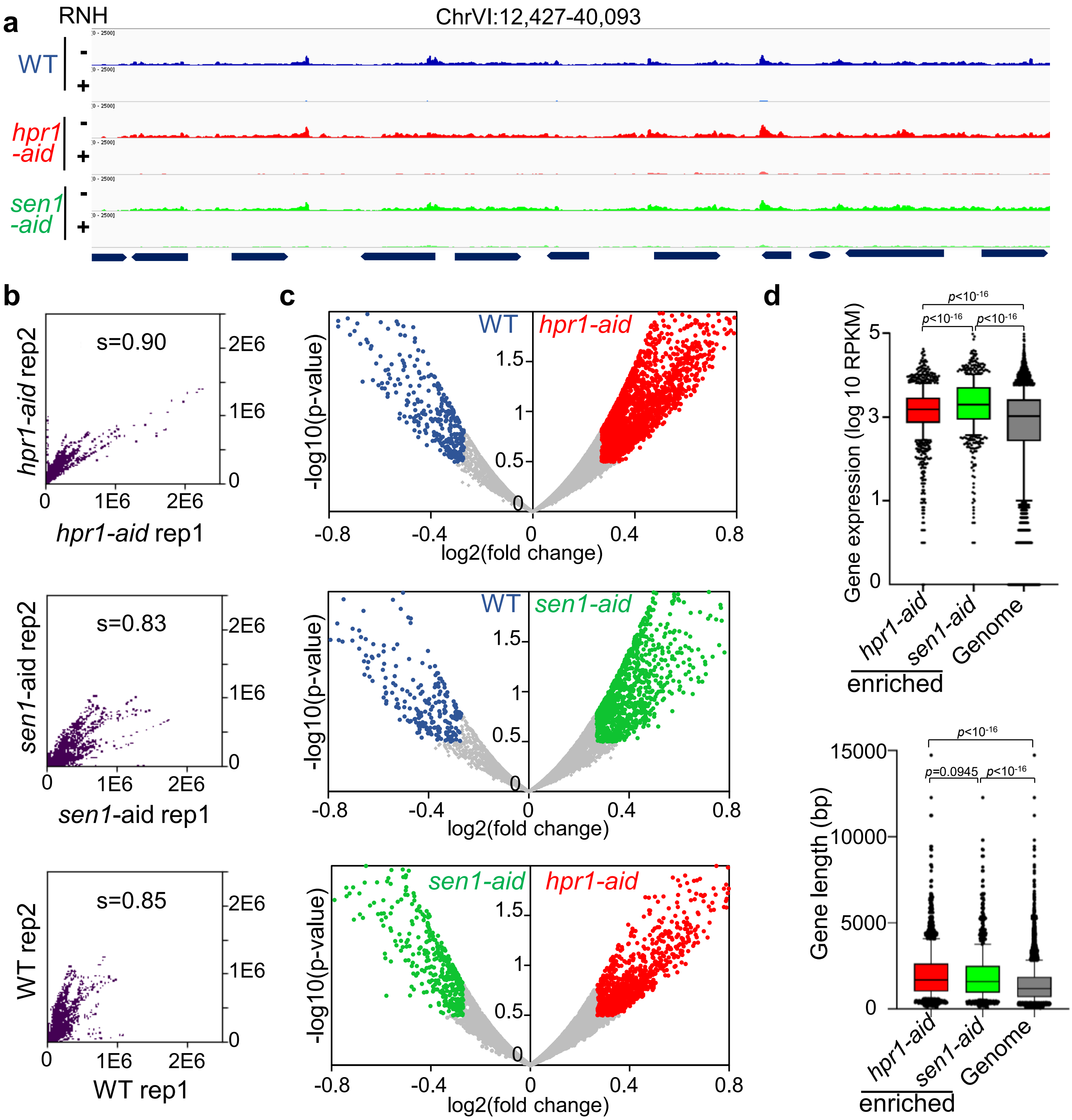

Supplementary Fig. 3. R-loop genome-wide accumulation after depletion of Hpr1 and Sen1 using an inducible auxin degron system. a. Representative screenshot of a genomic region in which DRIPc-seq signal from WT (blue), *hpr1-aid* (red) and *sen1-aid* (green) cells with or without RNH treatment is plotted. **b.** Correlation plot between the two DRIPc-seq replicas of *hpr1-aid* (top), *sen1-aid* (center) and WT (bottom). Spearman correlation coefficient is shown in each plot. **c.** Volcano plots showing the peaks differential enrichment between WT (blue) and *hpr1-aid* (red); WT and *sen1-aid* (green); and *hpr1-aid* and *sen1-aid* conditions. 1.2 fold-change and 0.6 –log10(p-value) were set as threshold. **d.** Boxplots showing gene expression (top) and gene length (bottom) distribution of *hpr1-aid* (red, n=1243 genes) and *sen1-aid* (green, n=778 genes) enriched genes, and all the protein coding genes of the genome (grey, n=6784 genes). The median of each pool is shown at the center of the box. Minimum and maximum values represent 10-90 percentile, respectively. Bounds of each box represent 25-75 percentile, respectively. The P values were calculated by the Mann-Whitney U-test, two tailed.

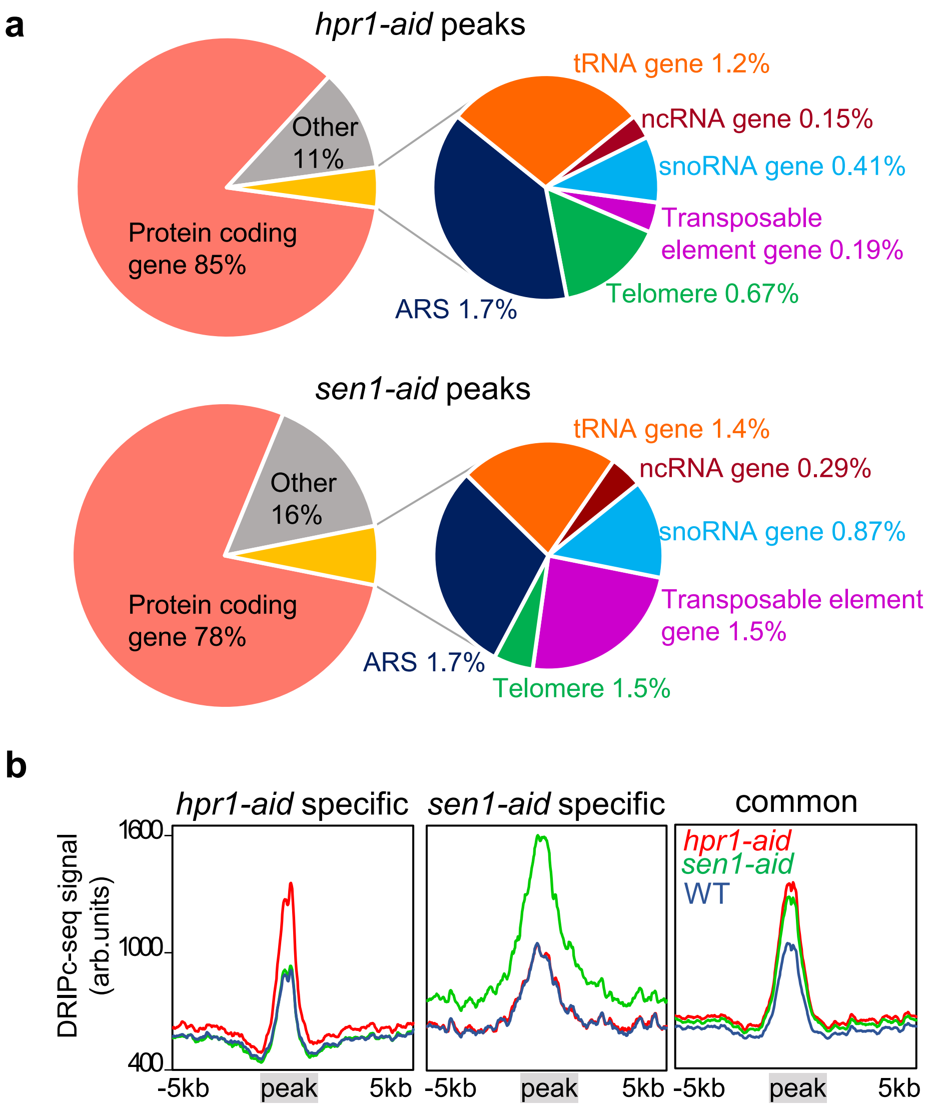

Supplementary Fig. 4. R-loop distribution after depletion of Hpr1 and Sen1 using an inducible auxin degron system. a. Increased R-loop peaks across several genomic features enriched in *hpr1-aid* and *sen1-aid* cells compared to WT. **b.** DRIPc-seq signal (average coverage, arbitrary units) metaplot analysis of ±2Kb of specific peaks in *hpr1-aid* and *sen1-aid* cells and common peaks respect to the WT. WT (blue), *hpr1-aid* (red) and *sen1-aid* (green).

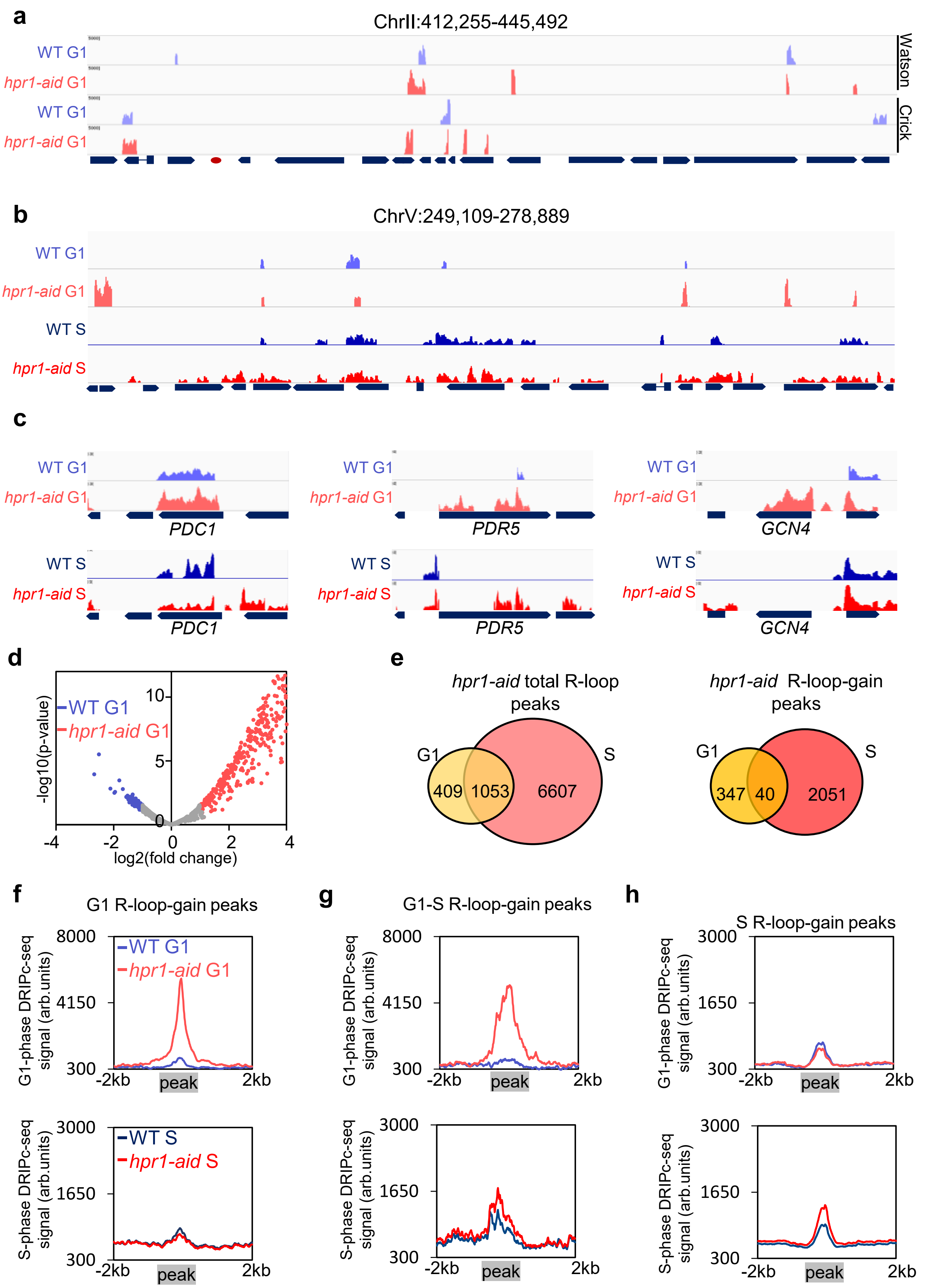
a
ChrII:412,255-445,492
WT G1
hpr1-aid G1
WT G1
hpr1-aid G1
Watson
Crick
b
ChrV:249,109-278,889
WT G1
hpr1-aid G1
WT S
hpr1-aid S
c
WT G1
hpr1-aid G1
PDC1
PDR5
GCN4
WT S
hpr1-aid S
d
-log10(p-value)
WT G1
hpr1-aid G1
log2(fold change)
e
hpr1-aid total R-loop peaks
G1
S
409
1053
6607
hpr1-aid R-loop-gain peaks
347
40
2051
f
G1 R-loop-gain peaks
G1-phase DRIPc-seq signal (arb.units)
8000
4150
300
-2kb
peak
2kb
S-phase DRIPc-seq signal (arb.units)
3000
1650
WT S
hpr1-aid S
g
G1-S R-loop-gain peaks
h
S R-loop-gain peaks

Supplementary Fig. 5. DNA-RNA hybrids genome wide accumulation after hpr1 depletion in G1 phase. a. Representative screenshot of a genomic region showing the DRIPc-seq signal of detected peaks profiles of G1 phase for WT (light blue) and *hpr1-aid* (pink) mapped at Watson and Crick strand. **b.** Representative screenshot of a genomic region showing the DRIPc-seq signal of detected peaks profiles for WT (light blue) and *hpr1-aid* (pink) in G1 phase, and WT (dark blue) and *hpr1-aid* (red) in S phase. **c.** Screenshots of *PDC1*, *PDR5* and *GCN4* loci showing the DRIPc-seq signal of detected peaks profiles of G1 phase (top) for WT (light blue) and hpr1-aid (pink), and signal of detected peaks profiles of S phase (bottom) for WT (dark blue) and *hpr1-aid* (red). **d.** Volcano plot showing the peaks differential enrichment between WT (light blue) and *hpr1-aid* (pink) G1 phase conditions. 2 fold-change and 1 -log10(p-value) were set as threshold. **e.** Venn diagrams showing the overlap between total R-loop peaks detected in hpr1-aid in G1 and S phases (left); and the overlap between the R-loop-gain peaks detected in *hpr1-aid* in G1 and S phases (right). **f.** DRIPc-seq signal (average coverage, arbitrary units) metaplot analysis of ±2kb G1 specific R-loop-gain peaks regions for WT (light blue) and *hpr1-aid* (pink) in G1 phase (top) and WT (dark blue) and *hpr1-aid* (red) in S phase (bottom). **g.** DRIPc-seq signal (average coverage, arbitrary units) metaplot analysis of ±2kb G1-S R-loop-gain peaks regions for WT (light blue) and *hpr1-aid* (pink) in G1 phase (top) and WT (dark blue) and *hpr1-aid* (red) in S phase (bottom). **h.** DRIPc-seq signal (average coverage, arbitrary units) metaplot analysis of ±2kb S specific R-loop-gain peaks regions for WT (light blue) and *hpr1-aid* (pink) in G1 phase (top) and WT (dark blue) and *hpr1-aid* (red) in S phase (bottom).

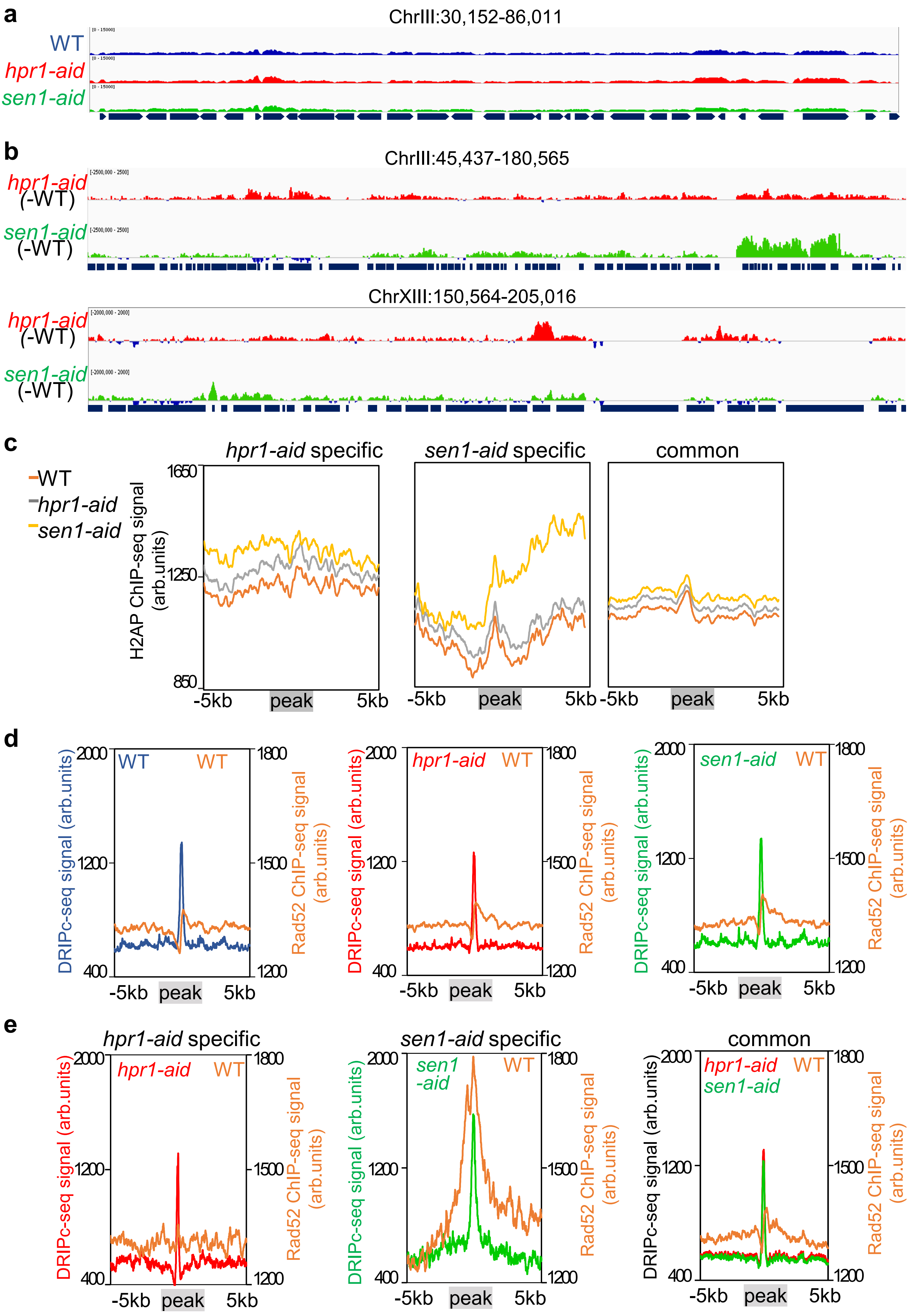
a
ChrIII:30,152-86,011
WT
hpr1-aid
sen1-aid
b
ChrIII:45,437-180,565
hpr1-aid (-WT)
sen1-aid (-WT)
ChrXIII:150,564-205,016
hpr1-aid (-WT)
sen1-aid (-WT)
c
WT
hpr1-aid
sen1-aid
H2AP ChIP-seq signal (arb.units)
hpr1-aid specific
sen1-aid specific
common
1650
1250
850
-5kb
peak
5kb
d
DRIPc-seq signal (arb.units)
Rad52 ChIP-seq signal (arb.units)
WT
hpr1-aid
sen1-aid
2000
1200
400
1800
1500
1200
-5kb
peak
5kb
e
hpr1-aid specific
sen1-aid specific
common
sen1 -aid
DRIPc-seq signal (arb.units)
Rad52 ChIP-seq signal (arb.units)
2000
1200
400
1800
1500
1200
-5kb
peak
5kb

Supplementary Fig. 6. Genome-wide H2AP distribution, and its correlation with R-loops after depletion of Hpr1 and Sen1 using an inducible auxin degron system. **a.** Representative screenshots of different genomic regions in which H2AP ChIP-seq signal from WT (blue), *hpr1-aid* (red) and *sen1-aid* (green) cells are shown. **b.** Representative screenshots of different genomic regions in which H2AP ChIP-seq signal from *hpr1-aid* (dark red) and *sen1-aid* (dark green) after subtracting the WT signal is shown. **c.** Metaplot analysis of H2AP ChIP-seq signal (average coverage, arbitrary units) surrounding DRIPc peaks ±5Kb of specific peaks in *hpr1-aid* and *sen1-aid* cells and common peaks respect to the WT, in WT (orange), *hpr1-aid* (grey) and *sen1-aid* (yellow) cells. **d.** Metaplot analysis of DRIPc-seq peaks (average coverage, arbitrary units) ±5Kb and WT Rad52 ChIP-seq signal (orange, average coverage, arbitrary units) in WT (blue), *hpr1-aid* (red) and *sen1-aid* (green) cells. **e.** Metaplot analysis of DRIPc-seq peaks (average coverage, arbitrary units) ±5Kb and WT Rad52 ChIP-seq signal (orange, average coverage, arbitrary units) of specific peaks in *hpr1-aid* and *sen1-aid* cells and common peaks respect to the WT, in WT (blue), *hpr1-aid* (red) and *sen1-aid* (green) cells.

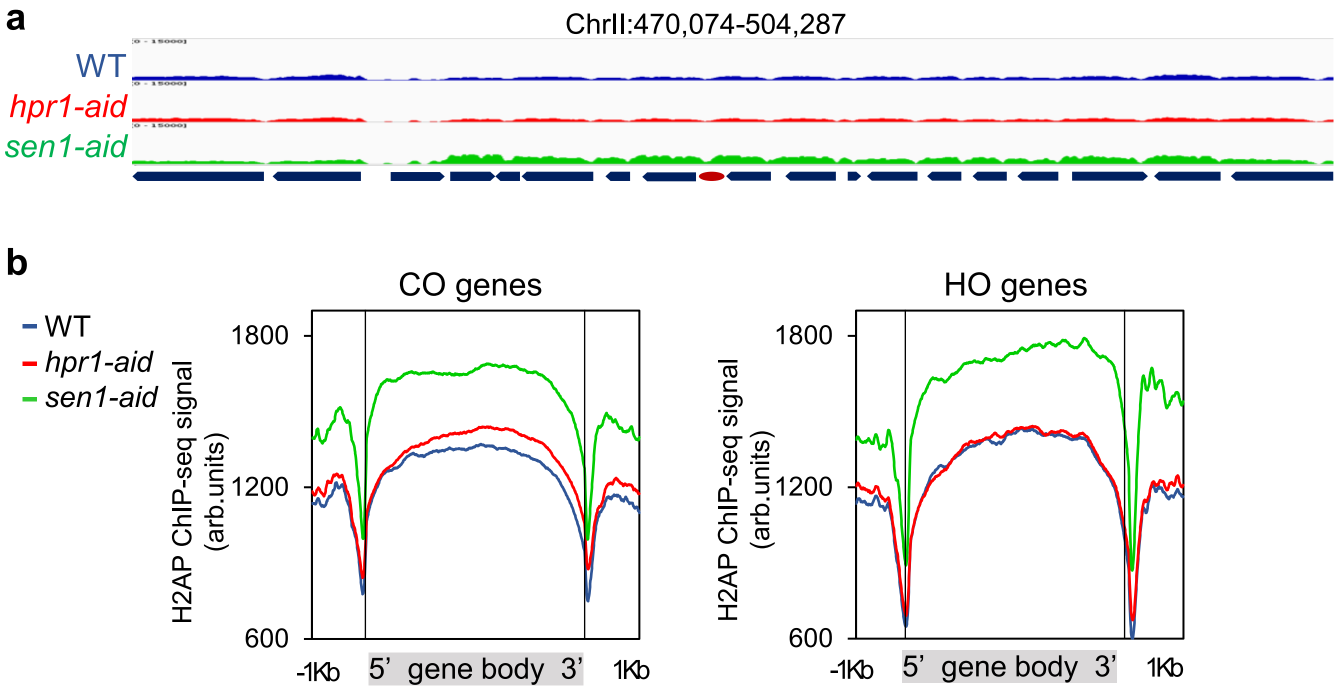

Supplementary Fig. 7. R-loop and H2AP distribution in early ARS in *hpr1* and *sen1* conditional mutants.
a. Representative screenshots of a genomic region containing an ARS in which H2AP ChIP-seq signal from WT (blue), *hpr1-aid* (red) and *sen1-aid* (green) cells are shown. **b.** Distribution of H2AP ChIP-seq signal (average coverage, arbitrary units) along HO and CD genes metaplot for WT (blue), *hpr1-aid* (red) and *sen1-aid* (green) cells.

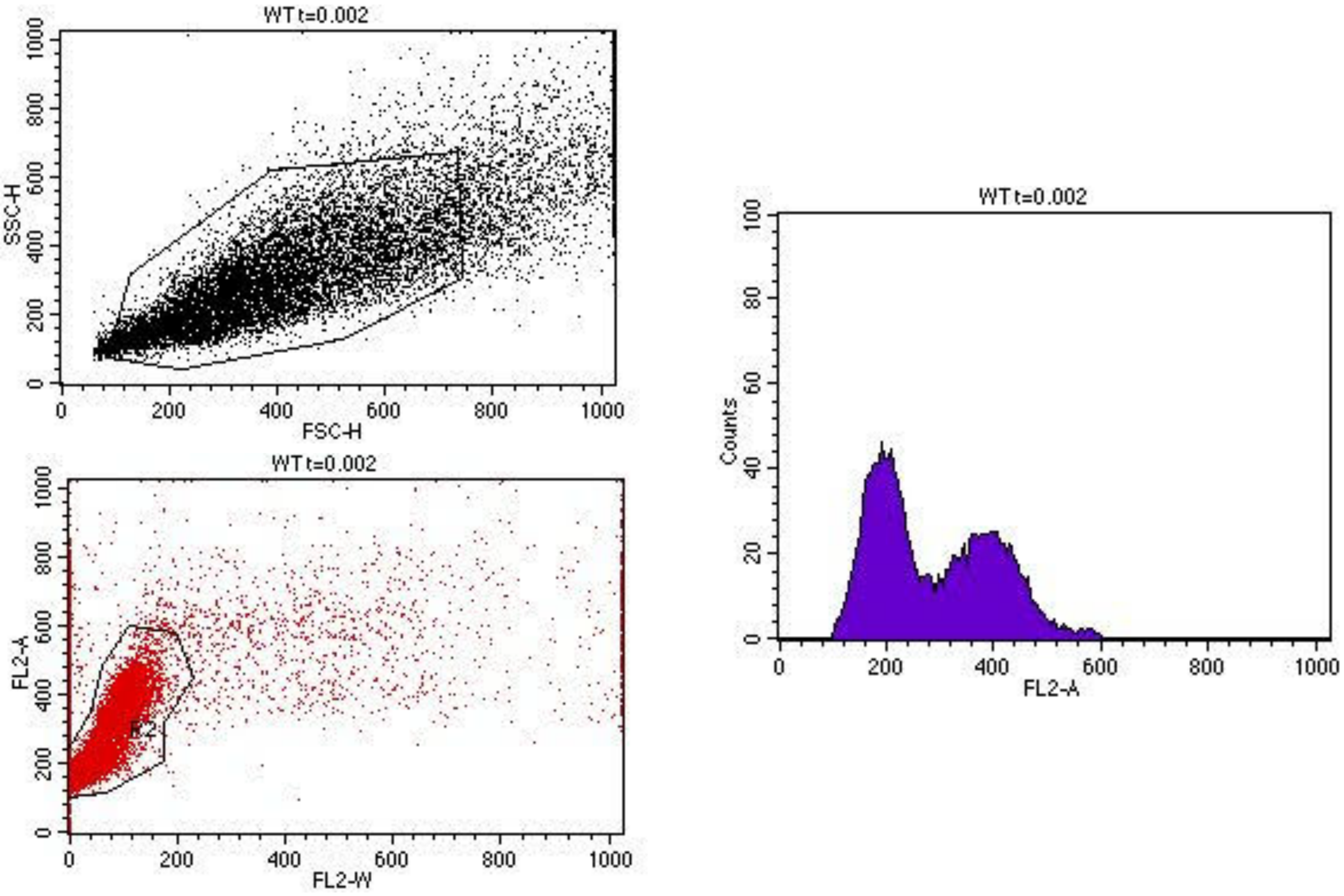

Supplementary Fig. 8. Gating strategy in flow cytometry experiments. Side scatter (SSC-A) and forward scatter (FSC-A) are plotted against each for two populations: smaller G2 and larger cells G1. Yeast cells were gated for size and propidium iodide (PI) intensity. PI analysis permitted to differentiate cell cycle stages, and yeast were sorted into the populations asynchronous, G1, and S. The same strategy was followed in the figures 2a, S2a, S2d and S2e.

FL2-A: total cell fluorescence , FL2-W: pulse width, SSC-H: side scatter.

Supplementary Table 1. Yeast strains used in this study.

Strain	Genotype	Source
F4	*MATa thr4*	G. Fink
F15	*MATα thr1 arg4*	G. Fink
Ybp249	*MATa ade2-1 can1-100 his3-11,15 leu2-3,112 trp1-1 ura3-1 bar1Δ RAD5*	Moriel-Carretero M. And Aguilera A., 2010
Ybp250	*MATα ade2-1 can1-100 his3-11,15 leu2-3,112 trp1-1 ura3-1 bar1Δ RAD5*	Moriel-Carretero M. And Aguilera A., 2010
YMK612	*MATa leu2-3,112 trp1-1 can1-100 ura3-1 ade2-1 his3-11,15 URA3-1::ADH1-AtTIR1-9Myc (URA3)*	Nishimura et al, 2009
hpr1-aid	YMK612 *MATa ura3-3*::*ADH1*-AtTIR1-9myc (*URA3*) *RAD5 bar1Δ hpr1::hpr1-aid (hphB)*	This study
hpr1-wt1	YMK612 *MATα ura3-3*::*ADH1*-AtTIR1-9myc (*URA3*) *RAD5 bar1Δ*	This study
hpr1-wt2	YMK612 *MATa ura3-3*::*ADH1*-AtTIR1-9myc (*URA3*) *RAD5 bar1Δ*	This study
sen1-aid	YMK612 *MATa ura3-3*::*ADH1*-AtTIR1-9myc (*URA3*) *RAD5 bar1Δ sen1::sen1-aid (hphB)*	This study
sen1-wt1	YMK612 *MATα ura3-3*::*ADH1*-AtTIR1-9myc (*URA3*) *RAD5 bar1Δ*	This study
sen1-wt2	YMK612 *MATa ura3-3*::*ADH1*-AtTIR1-9myc (*URA3*) *RAD5 bar1Δ*	This study
SEN1-R	*MATa leu2-3,112 trp1-1 can1-100 ura3-1 ade2-1 his3-11,15 sen1-1 RAD5 bar1Δ*	García-Pichardo et al, 2017
HPBAR1-R	*MATa leu2-3,112 trp1-1 can1-100 ura3-1 ade2-1 his3-11,15 bar1Δ hpr1ΔHIS3 RAD5*	This study
HPBAR1-R2	*MATα leu2-3,112 trp1-1 can1-100 ura3-1 ade2-1 his3-11,15 bar1ΔHyg hpr1ΔHIS3 RAD5*	This study
hpr1-d1 BrdU 7	YMK612 MATa ura3-3::ADH1-AtTIR1-9myc (*URA3*) *hpr1::hpr1-aid (hphB) RAD5 bar1Δ HIS3::BrdU-Inc*	This study
WT-d1 BrdU 1	YMK612 *MATa ura3-3::ADH1-At-TIR1-9myc (URA3) RAD5 HIS3::BrdU-Inc*	This study
sen1-d1 BrdU 2	YMK612 MATa ura3-3::ADH1-AtTIR1-9myc (*URA3*) *sen1::sen1-aid (hphB) RAD5 bar1Δ HIS3::BrdU-Inc*	This study

Supplementary Table 2. DNA primers used in standard PCR

Standard PCR		
Primer	Sequence 5' to 3'	Use
RAD5 UP	GCAGCAGGACCATGTAAACG	Check RAD5 strains
RAD5 LOW	AAACTCGTTACTCCACTGCG	Check RAD5 strains
HPR1 TAG DOWN	GAAGATAACAATGCAGCTACTTCGAACATTTCTAATGGTTC ATCTACCCAAGATATGAAA CGT ACG CTG CAG GTC GAC	Generate degron strains
HPR1 TAG UP	TCTCTGATAGGATCTCTGTGGTACGCTAAAATCTATCTGAA TTGTTTGGGACACTATGCA TCGATGAATTCGAGCTCG	Generate degron strains
comp HPR1 DOWN	GGAGTTTGCTCCACAGAATAC	Check degron strains
comp HPR1 UP	CTAAGGACGTTATTGACGCCTG	Check degron strains
SEN1 TAG DOWN	AGTTCGAGACGGAATGCTTCATCTAGCCCATTTATCCCAAA AAAAAGAAAGCCTAGATCA CGTACG CTGCAGGTCGAC	Generate degron strains
SEN1 TAG UP	AATAATGAAAAATAATGAAAAATAATGTTGCTATTAAATTCA ATATAAATGTATACACCTCGATGAATTCGAGCTCG	Generate degron strains
comp SEN1 DOWN	CCGCAACCACAATCTTCAGC	Check degron strains
comp SEN1 UP	GACTCCCTCATTCTTGGTTGC	Check degron strains
IAA (A) comp	AGAACGTGATGGTTTCCTGCC	Check degron strains
IAA (B) comp	TTTTGGCAGGAAACCATCACG	Check degron strains
BAR1 C	TTAGAGATGCGTTGTCCCTG	Generate *bar1*Δ strains
BAR1 D	TACGGACGTTTAGGATGACG	Generate *bar1*Δ strains
COMP BAR-HYG REV	GTCAGGCTCTCGCTGAATTC	Check *bar1*Δ strains
SONDA HYG FOR	GAC GTC TGT CGA GAA GTT TC	Check *bar1*Δ strains
KANB	CTGCAGCGAGGAGCCGTAAT	Check KAN resistant strains
NATF	GCCACTGAGGTTCTTCTTTC	Check NAT resistant strains
SONDA TK UP	GATGACTTACTGGCAGGTGC	TK probe
SONDA TK DOWN	ATGTGTCTGTCCTCCGGAAG	TK probe
RS1	TGAAAACCTCTGACACATGCAG	Primer sequences for confirmation of BrdU-Inc constructions
RS2	CTTGATTAGGGTGATGGTTCACG	Primer sequences for confirmation of BrdU-Inc constructions
5′His3	CTACTATTGCTTTGCTGTGGG	Primer sequences for confirmation of BrdU-Inc constructions
3′His3	GCCACCTATCACCACAACTAAC	Primer sequences for confirmation of BrdU-Inc constructions

Supplementary Table 3. RT-qPCR primers used in this study

RT-qPCR		
Primer	Sequence 5' to 3'	Use
GCN4 3' F	TTGTGCCCGAATCCAGTGA	ChIP/DRIP 3' region of the GCN4 gene
GCN4 3' R	TGGCGGCTTCAGTGTTTCTA	
PDR5 F	GTCAGAGGCTATATTTCACTGGAGAA	ChIP/DRIP middle region of the PDR5 gene
PDR5 R	TACGTCTTGTTTCGGCCTTAATC	
SPF1 5' UP	AGTCATTAATAGCAAAGCCGT	ChIP/DRIP 5' region of the SPF1 gene
SPF1 5' DOWN	GGTCCTTTGATGTAACGATCA	
SPF1 3` UP	CCCGTGGTAAACCTTTAGAAA	ChIP/DRIP 3' region of the SPF1 gene
SPF1 3' DOWN	ATATGAACGGCAAATTGAGAC	
ARS1211 REG3 FWD	GTTCCTCCACCTCCTTTGTGT	ChIP/DRIP region 3 of the PDC1 gene
ARS1211 REG3 REV	TGACCGATATATTGTGTTTCTATACTGTGT	
ARS1211 REG4 FWD	CGTTCAATTCGTTGGCGTTAC	ChIP/DRIP region 4 of the PDC1 gene
ARS1211 REG4 REV	TTAACACCGTTTTCGGTTTGC	
NR UP	TGCCTGCACGCCATTGT	ChIP/DRIP late replicative control region chromosome V
NR LOW	TTCCCCACGGAAAGTTGTATCT	
SCR1 UP	CGCACCGTGCCCTGTT	RT-PCR
SCR1 LOW	AGCTCTGCCCAGGACAAATTT	
HPR1 FW	TTTGAGGGAAAACCCGTTGA	RT-PCR
HPR1 RV	GCCCACGCTTTTTGTTAACTTC	

Supplementary Table 4. Plasmids used in this study.

Name	Description	Source
pWJ1344	YCp containing the RAD52::YFP fusion (*LEU* marker)	Lisby et al. 2001
pSCH204	YCp prs314-LB containing the *L-LacZ* recombination system under the LEU promoter with the 3 kb fragment BamHI from *LacZ* inserted between the leu2 direct repeats	Chávez and Aguilera. 1997
p403-BrdU-Inc	pRS403 containing the BrdU-Inc cassette (*ADH1*-hENT1 and GDP-HSV-TK constructions allowing BrdU incorporation)	Aparicio O. M. And Viggiani C.J. 2006
pRS313	YCp vector based on the *HIS3* marker.	Hieter P. 1989
pRS313-GALRNH1	YCp containing the *RNH1* gene under *GAL* promoter (*HIS3* marker)	García-Benítez et al. 2017
pRS414-GAL1	YCp vector based on the *TRP1* marker.	Funk M. 1994
pCM184	Centromeric plasmid containing Tet promoter and *TRP1* marker.	Gari et al. 1997
pCM184RNH1	*RNH1* ORF cloned into pCM184 in NotI site	Santos-Pereira et al. 2013
pHyg-AID*-9myc	pSM409 vector containing AID tag with the selection marker hphNT1 and the extension 9myc used to generate degron strains.	Morawska and Ulrich 2013

Supplementary Table 5. Antibodies used in this study

Antibodies			
Name	Use	Source	Identifier
Mouse monoclonal c-Myc (Clone 9E10)	WB (1:500)	Sigma-Aldrich	Cat# M4439; RRID:AB_627268
Mouse monoclonal anti-actin	WB (1:2000)	Abcam	Cat# ab8224; RRID:AB_449644
Rabbit polyclonal anti-actin	WB (1:2000)	Abcam	Cat# ab8227; RRID:AB_2305186
Rabbit polyclonal anti-H2A (Ser129)	ChIP (5 µl)	Abcam	Cat# ab15083; RRID:AB_301630
Rabbit polyclonal anti-H2A (Ser129)	WB (1:1000)	Active motif	Cat# 39235; RRID:AB_2687477
Mouse monoclonal anti-BrdU	ChIP (3 µl)	MBL	Cat# MI-11-3; RRID:AB_590678
IRDye 800CW Goat anti-Rabbit IgG (H+L)	WB (1:15000)	LI-COR Biosciences	Cat# 925-32211; RRID AB_2651127
IRDye 680RD Goat anti-mouse IgG (H+L)	WB (1:15000)	LI-COR Biosciences	Cat# 926-68070; RRID AB_10956588
Alexa Fluor 555 Goat anti-mouse IgG (H+L)	IF (1:1000)	Thermo Fisher Scientific	Cat# A-21424; RRID AB_141780
S9.6 antibody	IF (1:300)<br>DRIP (3 µl)	Homemade	
