# Supplementary material for: Harmful R-loops are prevented via different cell cycle-specific mechanisms
Source: Nat Commun. 2021 Jul 22;12:4451. doi: 10.1038/s41467-021-24737-x (PMC8298424; doi:10.1038/s41467-021-24737-x)
Supplement: Supplementary file 3 — Reporting Summary [file 41467_2021_24737_MOESM3_ESM.pdf]

nature research

Corresponding author(s): Andres Aguilera
Tatiana García-Muse

Last updated by author(s): Apr 21, 2021

Reporting Summary

Nature Research wishes to improve the reproducibility of the work that we publish. This form provides structure for consistency and transparency in reporting. For further information on Nature Research policies, see our Editorial Policies and the Editorial Policy Checklist.

Statistics

For all statistical analyses, confirm that the following items are present in the figure legend, table legend, main text, or Methods section.

n/a	Confirmed	
☐	☒	The exact sample size (n) for each experimental group/condition, given as a discrete number and unit of measurement
☐	☒	A statement on whether measurements were taken from distinct samples or whether the same sample was measured repeatedly
☐	☒	The statistical test(s) used AND whether they are one- or two-sided<br>*Only common tests should be described solely by name; describe more complex techniques in the Methods section.*
☒	☐	A description of all covariates tested
☐	☒	A description of any assumptions or corrections, such as tests of normality and adjustment for multiple comparisons
☐	☒	A full description of the statistical parameters including central tendency (e.g. means) or other basic estimates (e.g. regression coefficient) AND variation (e.g. standard deviation) or associated estimates of uncertainty (e.g. confidence intervals)
☐	☒	For null hypothesis testing, the test statistic (e.g. F, t, r) with confidence intervals, effect sizes, degrees of freedom and P value noted<br>*Give P values as exact values whenever suitable.*
☒	☐	For Bayesian analysis, information on the choice of priors and Markov chain Monte Carlo settings
☒	☐	For hierarchical and complex designs, identification of the appropriate level for tests and full reporting of outcomes
☒	☐	Estimates of effect sizes (e.g. Cohen's d, Pearson's r), indicating how they were calculated

Our web collection on statistics for biologists contains articles on many of the points above.

Software and code

Policy information about availability of computer code

Data collection	Microscopy images were acquired the with a Leica DM6000 microscope equipped with a DFC390 camera and LAS AX v2.0 image acquisition software (Leica).<br>Real-time quantitative PCRs (qPCRs) were performed on a 7500 Fast Real-Time PCR system (Applied Biosystems, Carlsbad, CA).<br>Quantitative PCR results were obtained from a 7500 FAST Real-Time PCR System equipped with 7500 Software v2.3.<br>Flow cytometry analysis were performed using a BFACScalibur (Becton Dickison fluorescence-activated cell analyzer) and analyzed by BD CellQuest Pro Software v5.1.
Data analysis	Western blots bands were quantified using ImageStudio software v3.1 (LI-COR biosciences).<br>Display of plots and statistical analyses were carried out using the Prism software (GraphPad) v8.<br>Images were processed using Adobe Photoshop CS4.<br>For genome wide data, sequenced paired-ends reads were subjected to quality control pipeline using the FASTQ Toolkit V.1.0.0 software (Illumina) and then mapped to the Saccharomyces cerevisiae reference genome using the Rsubread V 2.0.1 software package. Mapped reads were assigned to Watson or Crick strand using SAMtools V 1.1072. Peak calling was performed with chromstaR V 1.12.0 software package43. For comparative analysis, regions covered by peaks in the two conditions that are being compared were merged and fused when closer than 200 bp distance using BEDtools V 2.27.173. The differential enrichment of these regions in each condition was performed using csaw V 1.20.0 software package74. After that, edgeR package (v3.20.9) was used in order to calculate log2FC and p-value of the peaks. Coverage profiling were obtained using bamCoverage tool from deepTools V 3.4.378. Genome example regions were plotted using IGV V 2.8.2 software79.

For manuscripts utilizing custom algorithms or software that are central to the research but not yet described in published literature, software must be made available to editors and reviewers. We strongly encourage code deposition in a community repository (e.g. GitHub). See the Nature Research guidelines for submitting code & software for further information.

Data

Policy information about availability of data

All manuscripts must include a data availability statement. This statement should provide the following information, where applicable:

- Accession codes, unique identifiers, or web links for publicly available datasets
- A list of figures that have associated raw data
- A description of any restrictions on data availability

The DRIPc-seq and H2AP ChIP-seq data generated are available at NCBI's Sequence Read Archive (SRA) under accession number GSE159870 [https://www.ncbi.nlm.nih.gov/geo/query/acc.cgi?acc=GSE159870]. This dataset was used in figures 5, 6, S3, S4, S5, S6 and S7.
The Rad52 ChIP-seq data from doi: 10.1016/j.molcel.2018.06.037 are available at NCBI's Sequence Read Archive (SRA) under accession number GSE110575 [https://www.ncbi.nlm.nih.gov/geo/query/acc.cgi?acc=GSE110575]. This dataset was used in figure S3.
The RNA-seq data from https://doi.org/10.1186/s13104-019-4286-0 are available at NCBI's Sequence Read Archive (SRA) under accession number SAMN11070697 [[https://www.ncbi.nlm.nih.gov/biosample/SAMN11070697].
Thisdataset was used in figure S6.
Genomic features coordinates used are available at Saccharomyces Genome Database (SGD) [https://www.yeastgenome.org/].
Early ARS coordinates are available at DOI: 10.1186/1471-2164-15-791, and have been used in figure 6

Field-specific reporting

Please select the one below that is the best fit for your research. If you are not sure, read the appropriate sections before making your selection.

☒ Life sciences ☐ Behavioural & social sciences ☐ Ecological, evolutionary & environmental sciences

For a reference copy of the document with all sections, see nature.com/documents/nr-reporting-summary-flat.pdf

Life sciences study design

All studies must disclose on these points even when the disclosure is negative.

Sample size	All experiments were carried out with yeast cells with multiple available biological replicates and based on previous experience and similar experiments on previous publications such as García-Pichardo D, Cañas JC, García-Rubio ML, Gómez-González B, Rondón AG, Aguilera A. Histone Mutants Separate R Loop Formation from Genome Instability Induction. Mol Cell. 2017 Jun 1;66(5):597-609.e5. doi: 10.1016/j.molcel.2017.05.014. PMID: 28575656, Herrera-Moyano E, Mergui X, García-Rubio ML, Barroso S, Aguilera A. The yeast and human FACT chromatin-reorganizing complexes solve R-loop-mediated transcription-replication conflicts. Genes Dev. 2014 Apr 1;28(7):735-48. doi: 10.1101/gad.234070.113. Epub 2014 Mar 17. PMID: 24636987; PMCID: PMC4015491, Lafuente-Barquero J, García-Rubio ML, Martin-Alonso MS, Gómez-González B, Aguilera A. Harmful DNA:RNA hybrids are formed in cis and in a Rad51-independent manner. Elife. 2020;9:e56674. Published 2020 Aug 4. doi:10.7554/eLife.56674.
Data exclusions	No samples were excluded.
Replication	All experiments were conducted in several replicates as indicated in the figure legends.
Randomization	Samples were not allocated into groups.
Blinding	In microscopy analysis, the same settings were used in data collection and data analysis. In all the experiments, blinding was not possible as data collection and analysis was performed by the same person.

Reporting for specific materials, systems and methods

We require information from authors about some types of materials, experimental systems and methods used in many studies. Here, indicate whether each material, system or method listed is relevant to your study. If you are not sure if a list item applies to your research, read the appropriate section before selecting a response.

Materials & experimental systems

n/a	Involved in the study	
☐	☒	Antibodies
☒	☐	Eukaryotic cell lines
☒	☐	Palaeontology and archaeology
☒	☐	Animals and other organisms
☒	☐	Human research participants
☒	☐	Clinical data
☒	☐	Dual use research of concern

Methods

n/a	Involved in the study	
☐	☒	ChIP-seq
☐	☒	Flow cytometry
☒	☐	MRI-based neuroimaging

Antibodies

Antibodies used

All the antibodies used in this study were company-validated antibodies. Catalog numbers and RRID are provided to facilitate access to all the information regarding their validation.
Mouse monoclonal c-Myc (Clone 9E10) Sigma-Aldrich Cat# M4439; RRID:AB_627268
Mouse monoclonal anti-actin Abcam Cat# ab8224 clone [mAbcam 8224]; RRID:AB_449644
Rabbit polyclonal anti-actin Abcam Cat# ab8227; RRID:AB_2305186
Rabbit polyclonal anti-H2A (Ser129) Abcam Cat# ab15083; RRID:AB_301630
Rabbit polyclonal anti-H2A (Ser129) Active motif Cat# 39235; RRID:AB_2687477
Mouse monoclonal anti-BrdU MBL Cat# MI-11-3 (Clone 2B1); RRID:AB_590678
IRDye 800CW Goat anti-Rabbit IgG (H+L) LI-COR Biosciences Cat# 925-32211; RRID AB_2651127
IRDye 680RD Goat anti-mouse IgG (H+L) LI-COR Biosciences Cat# 926-68070; RRID AB_10956588
Alexa Fluor 555 Goat anti-mouse IgG (H+L) Thermo Fisher Scientific Cat# A-21424; RRID AB_141780
S9.6 antibody homemade (use 1:300).

Validation

For S9.6 antibody, signals were significantly removed after RNase H t reatment which specifically digests RNA/DNA hrbrids (Fig 1e, 1f, S3a). This antibody was previously shown to bind dsRNA (Hartono et al., 2018). We pretreated samples with RNase A which digests RNAs in DRIP and DRIPc-seq experiments and RNase H which eliminates dsRNA in DRIPc-seq experiments.

Mouse monoclonal c-Myc (Clone 9E10) Sigma-Aldrich Cat# M4439; RRID:AB_627268. Validated in: Ambrosio S, Amente S, Napolitano G, Di Palo G, Lania L, Majello B. MYC impairs resolution of site-specific DNA double-strand breaks repair. Mutat Res. 2015 Apr;774:6-13. doi: 10.1016/j.mrfmmm.2015.02.005. Epub 2015 Mar 4. PMID: 25770827, Kim T, Jeon YJ, Cui R, Lee JH, Peng Y, Kim SH, Tili E, Alder H, Croce CM. Role of MYC-regulated long noncoding RNAs in cell cycle regulation and tumorigenesis. J Natl Cancer Inst. 2015 Feb 6;107(4):dju505. doi: 10.1093/jnci/dju505. PMID: 25663692; PMCID: PMC4402359.

Mouse monoclonal anti-actin Abcam Cat# ab8224 clone [mAbcam 8224]; RRID:AB_449644. Validated in: Jia R, Yi Y, Liu J, Pei D, Hu B, Hao H, Wu L, Wang Z, Luo X, Lu Y. Cyclic compression emerged dual effects on the osteogenic and osteoclastic status of LPS-induced inflammatory human periodontal ligament cells according to loading force. BMC Oral Health. 2020 Jan 6;20(1):7. doi: 10.1186/s12903-019-0987-y. PMID: 31907038; PMCID: PMC6945767, Zhang R, Wang J, Jia E, Zhang J, Liu N, Chi C. lncRNA BCAR4 sponges miR-370-3p to promote bladder cancer progression via Wnt signaling. Int J Mol Med. 2020 Feb;45(2):578-588. doi: 10.3892/ijmm.2019.4444. Epub 2019 Dec 27. PMID: 31894304; PMCID: PMC6984777.

Rabbit polyclonal anti-actin Abcam Cat# ab8227; RRID:AB_2305186. Validated in: Yang KL, Khoo BY, Ong MT, Yoong ICK, Sreeramanan S. In vitro anti-breast cancer studies of LED red light therapy through autophagy. Breast Cancer. 2021 Jan;28(1):60-66. doi: 10.1007/s12282-020-01128-6. Epub 2020 Jul 11. PMID: 32654094.

Rabbit polyclonal anti-H2A (Ser129) Abcam Cat# ab15083; RRID:AB_301630. Validated in: Chong SY, Cutler S, Lin JJ, Tsai CH, Tsai HK, Biggins S, Tsukiyama T, Lo YC, Kao CF. H3K4 methylation at active genes mitigates transcription-replication conflicts during replication stress. Nat Commun. 2020 Feb 10;11(1):809. doi: 10.1038/s41467-020-14595-4. PMID: 32041946; PMCID: PMC7010754, Kuo CH, Leu YL, Wang TH, Tseng WC, Feng CH, Wang SH, Chen CC. A novel DNA repair inhibitor, diallyl disulfide (DADS), impairs DNA resection during DNA double-strand break repair by reducing Sae2 and Exo1 levels. DNA Repair (Amst). 2019 Oct;82:102690. doi: 10.1016/j.dnarep.2019.102690. Epub 2019 Aug 24. PMID: 31479843.

Rabbit polyclonal anti-H2A (Ser129) Active motif Cat# 39235; RRID:AB_2687477. Validated in: Lei B, Capella M, Montgomery SA, Borg M, Osakabe A, Goiser M, Muhammad A, Braun S, Berger F. A Synthetic Approach to Reconstruct the Evolutionary and Functional Innovations of the Plant Histone Variant H2A.W. Curr Biol. 2021 Jan 11;31(1):182-191.e5. doi: 10.1016/j.cub.2020.09.080. Epub 2020 Oct 22. PMID: 33096036.

Mouse monoclonal anti-BrdU MBL Cat# MI-11-3 (Clone 2B1); RRID:AB_590678. Validated in: Katou Y, Kanoh Y, Bando M, Noguchi H, Tanaka H, Ashikari T, Sugimoto K, Shirahige K. S-phase checkpoint proteins Tof1 and Mrc1 form a stable replication-pausing complex. Nature. 2003 Aug 28;424(6952):1078-83. doi: 10.1038/nature01900. PMID: 12944972.

RRDye 800CW Goat anti-Rabbit IgG (H+L) LI-COR Biosciences Cat# 925-32211; RRID AB_2651127. Validated in Zhao S, Li W, Cheng F, Rao T, Yu W, Ruan Y, Yuan R, Yao X and Ning J: High-pressure carbon dioxide pneumoperitoneum induces oxidative stress and mitochondria-associated apoptotic pathway in rabbit kidneys with severe hydronephrosis. Int J Mol Med 43: 305-315, 2019.

IRDye 680RD Goat anti-mouse IgG (H+L) LI-COR Biosciences Cat# 926-68070; RRID AB_10956588. Validated in: Jordan, J., Chhim, S., Margulies, C. et al. ALKBH7 drives a tissue and sex-specific necrotic cell death response following alkylation-induced damage. Cell

Death Dis 8, e2947 (2017). https://doi.org/10.1038/cddis.2017.343.

Alexa Fluor 555 Goat anti-mouse IgG (H+L) Thermo Fisher Scientific Cat# A-21424; RRID AB_141780. Validated in:
Gao Z, Zhang H, Hu F, Yang L, Yang X, Zhu Y, Sy MS, Li C. Glycan-deficient PrP stimulates VEGFR2 signaling via glycosaminoglycan. Cell Signal. 2016 Jun;28(6):652-62. doi: 10.1016/j.cellsig.2016.03.010. Epub 2016 Mar 19. PMID: 27006333.

ChIP-seq

Data deposition

☒ Confirm that both raw and final processed data have been deposited in a public database such as GEO.

☐ Confirm that you have deposited or provided access to graph files (e.g. BED files) for the called peaks.

Data access links<br>*May remain private before publication.*	GSE159870.
Files in database submission	*Provide a list of all files available in the database submission.*
Genome browser session<br>(e.g. UCSC)	*Provide a link to an anonymized genome browser session for "Initial submission" and "Revised version" documents only, to enable peer review. Write "no longer applicable" for "Final submission" documents.*

Methodology

Replicates	*Describe the experimental replicates, specifying number, type and replicate agreement.*
Sequencing depth	*Describe the sequencing depth for each experiment, providing the total number of reads, uniquely mapped reads, length of reads and whether they were paired- or single-end.*
Antibodies	Rabbit polyclonal anti-H2A (Ser129) Abcam Cat# ab15083; RRID:AB_301630
Peak calling parameters	*Specify the command line program and parameters used for read mapping and peak calling, including the ChIP, control and index files used.*
Data quality	*Describe the methods used to ensure data quality in full detail, including how many peaks are at FDR 5% and above 5-fold enrichment.*
Software	*Describe the software used to collect and analyze the ChIP-seq data. For custom code that has been deposited into a community repository, provide accession details.*

Flow Cytometry

Plots

Confirm that:

☒ The axis labels state the marker and fluorochrome used (e.g. CD4-FITC).

☒ The axis scales are clearly visible. Include numbers along axes only for bottom left plot of group (a 'group' is an analysis of identical markers).

☐ All plots are contour plots with outliers or pseudocolor plots.

☒ A numerical value for number of cells or percentage (with statistics) is provided.

Methodology

Sample preparation	For FACS analysis, each yeast culture was centrifuged and washed with sodium citrate 50 mM pH 7.5 and resuspended in 1 ml of ethanol 70%. The samples could be stored at 4ºC until processing. Cells were washed once with sodium citrate 50 mM pH 7.5 and incubated 1 hour at 50ºC with 25 µl RNase A (10mg/ml). 50 µl of 20 mg/ml proteinase K was added and samples were incubated 1 hour at 50ºC. 1 ml of sodium citrate containing 16 µg/ml propidium iodide was added and samples were sonicated until cells were completely disaggregated. The samples were stored 30 minutes in the dark or 12-48 hours at 4ºC before the analysis. Before the flow cytometry, cells were sonicated 5 seconds at 10% amplitude and scored in FACScalibur (Becton Dickison fluorescence-activated cell analyzer). For each histogram 100000 yeast cells were analyzed.
Instrument	FACScalibur (Becton Dickison fluorescence-activated cell analyzer).
Software	BD CellQuest Pro Software v5.1.

Cell population abundance | 100000

Gating strategy | Yeast cells were gated for size sorted by propidium iodide (PI) intensity. PI analysis permitted to differentiate cell cycle stages, and yeast were sorted into the populations asynchronous, G1, and S. The same strategy was followed in the figures 2a, S2a, S2d and S2e.

☒ Tick this box to confirm that a figure exemplifying the gating strategy is provided in the Supplementary Information.
